# PATIENT-FOCUSED TELEHEALTH 101: CONTENT, RESULTS, FEEDBACK, AND PLANS FOR REVISION AND EXPANSION

**DOI:** 10.1093/geroni/igad104.0477

**Published:** 2023-12-21

**Authors:** Paul Freddolino, Ha Neul Kim, Fei Sun, Megan Bentley, Catherine Macomber

**Affiliations:** Michigan State University, East Lansing, Michigan, United States; Michigan State University, East Lansing, Michigan, United States; Michigan State University, East Lansing, Michigan, United States; Michigan State University, East Lansing, Michigan, United States; Saginaw Valley State University, University Center, Michigan, United States

## Abstract

A five-part patient-focused Telehealth 101 mini-course was offered one-to-one as part of a technology literacy program for older adults in a rural Midwest community who received home-delivered meals. Participants had already received content on basic digital skills including safety and security, email, internet browsing, videoconferencing, and photo tools. Topics included an overview of material to be covered, types of telehealth (real time encounters, remote patient monitoring, and patient portals), what to expect from providers, how to prepare for telehealth encounters, and a live simulation of a telehealth visit. Subjects rated the sessions positively, and patient engagement in health care (Patient Activation Measure scores) significantly increased after the training. The mini course was also offered virtually on Zoom to groups of older adults at five urban senior centers. Feedback from both settings suggest the need to reduce repetition in the material presented and to include more diversity in the personnel presenting the content, both live and through YouTube videos accessed as part of the content. Revision will also add content on mental health and substance abuse services available through telehealth.

